# Numerical investigations of MRI RF field induced heating for external fixation devices

**DOI:** 10.1186/1475-925X-12-12

**Published:** 2013-02-09

**Authors:** Yan Liu, Jianxiang Shen, Wolfgang Kainz, Songsong Qian, Wen Wu, Ji Chen

**Affiliations:** 1 University of Houston, Houston, TX, 77204, USA; 2 U.S. Food and Drug Administration, Silver Spring, MD, 20993, USA; 3 Nanjing University of Science and Technology, Nanjing, 210094, China

**Keywords:** RF heating, External fixation device, MRI compatible devices

## Abstract

**Background:**

The magnetic resonance imaging (MRI) radio frequency (RF) field induced heating on external fixation devices can be very high in the vicinity of device screws. Such induced RF heating is related to device constructs, device placements, as well as the device insertion depth into human subjects. In this study, computational modeling is performed to determine factors associated with such induced heating.

**Methods:**

Numerical modeling, based on the finite-difference time-domain (FDTD) method, is used to evaluate the temperature rises near external device screw tips inside the ASTM phantom for both 1.5-T and 3-T MRI systems. The modeling approach consists of 1) the development of RF coils for 1.5-T and 3-T, 2) the electromagnetic simulations of energy deposition near the screw tips of external fixation devices, and 3) the thermal simulations of temperature rises near the tips of these devices.

**Results:**

It is found that changing insertion depth and screw spacing could largely affect the heating of these devices. In 1.5-T MRI system, smaller insertion depth and larger pin spacing will lead to higher temperature rise. However, for 3-T MRI system, the relation is not very clear when insertion depth is larger than 5 cm or when pin spacing became larger than 20 cm. The effect of connection bar material on device heating is also studied and the heating mechanism of the device is analysed.

**Conclusions:**

Numerical simulation is used to study RF heating for external fixation devices in both 1.5-T and 3-T MRI coils. Typically, shallower insertion depth and larger pin spacing with conductive bar lead to higher RF heating. The heating mechanism is explained using induced current along the device and power decay inside ASTM phantom.

## Background

In magnetic resonance imaging (MRI) systems, strong electromagnetic fields at 64 MHz and 128 MHz are generated by RF coils for 1.5-T and 3-T MRI systems [1,2]. When patients with metallic implantable medical devices are undergoing such a procedure, the induced RF energy can generate localized energy deposition near these devices causing very high local temperature rise [3]. Accordingly, MRI RF induced heating needs to be assessed for such devices to ensure patient safety according to recommendations from the Food and Drug Administration (FDA). These recommendations are based on those appropriate test procedures presented by the American Society for Testing and Materials (ASTM) International [4]. Meanwhile, the time-varying magnetic field generated by gradient coils can also induce eddy currents on device according to Faraday’s law [5] and induce heating of metallic implants [5,6]. This research focused on heating effect generated from RF fields in MRI systems.

Numerical simulations and bench measurements have been used to evaluate the RF induced heating effect for various medical devices. These devices can be divided into two categories: active medical devices and passive medical devices. Active medical devices such as pacemakers and implantable neurological pulse generators (IPGs) have been studied extensively since these devices are implanted inside vital parts of the human body, such as heart muscle or brain. Kainz et al. [7] reported a maximum of 2.1°C temperature increases at the lead tip for neurological pulse generators. However, some other studies found much higher temperature rise which could be harmful to humans. Pisa et al. [8] found temperature increments from 0.6°C to 15°C for 6-min MRI investigation using a thorax model. Mattei et al. [9] studied the induced heating on metallic leads by measuring 375 experimental configurations and demonstrated the locations of the leads in the phantom and the lead structures have significant influence on pacemaker leads tip heating. Passive implanted devices have also been studied using both numerical and experimental methods. Liu et al. [10] used electromagnetic and thermal simulations to determine the worst-case heating for a spinal basis orthopedic implant with length variation from 21–107 mm combined with an experimental measurement for validation. Kumar et al. [11] studied the RF heating for 3 different orthopaedic devices and observed a maximum temperature rise of 1.5°C after two sequential 30-min exposures in MRI environment. Muranaka et al. [12] measured the RF heating on hip joint implant in 1.5-T MRI. Temperature rise of 9.0°C for the Co-Cr implant and 5.3°C for titanium implant are observed. Recently, there has been a growing interest in evaluating the MRI induced heating due to external fixation devices [13,14] where only parts of the devices are inside the human body. Compared to typical implantable devices where all devices are implanted inside human subjects, the external fixation devices have a significant part of the components external to the body. Since the electric field outside the human subject can be higher than that inside human body (due to no body tissue related attenuation), the potential heating for external fixation devices could be much higher. In some MRI labelling, the external fixation devices can lead to temperature rise up to 9.9°C temperature rise. Consequently, patients with such devices are currently not recommended for an MRI scan.

In orthopaedic surgery, the exact construct of an external fixation device needs to be adjusted to fit shapes and sizes of patient populations. A typical device is composed of bars, pins and clamps. Often, large body sizes require large pin spacing as well as deep pin insertion depth to achieve a good fixation or alignment. As a result, different patients may have different device configurations. Therefore, the effect of different insertion depth and pin spacing on device tip heating shall be investigated. In most practices, the connecting bars are made of metal to retain mechanical strength. Recently, other materials, such as carbon-fiber and plastic glass have been proposed/tested for such purposes. Therefore, the effects of using different connecting bar in MRI heating shall also be investigated. In this work, a comprehensive study of the external fixation construct parameters on the effect of MRI RF induced heating will be performed to understand the heating mechanism.

## Methods

In this numerical investigation, the modeling/simulation procedure consists of the following four parts: (1) the development of the transmit RF coil, (2) the appropriate placement of device inside the ASTM phantom, (3) the electromagnetic simulations, and (4) the thermal simulations.

**Figure 1:**
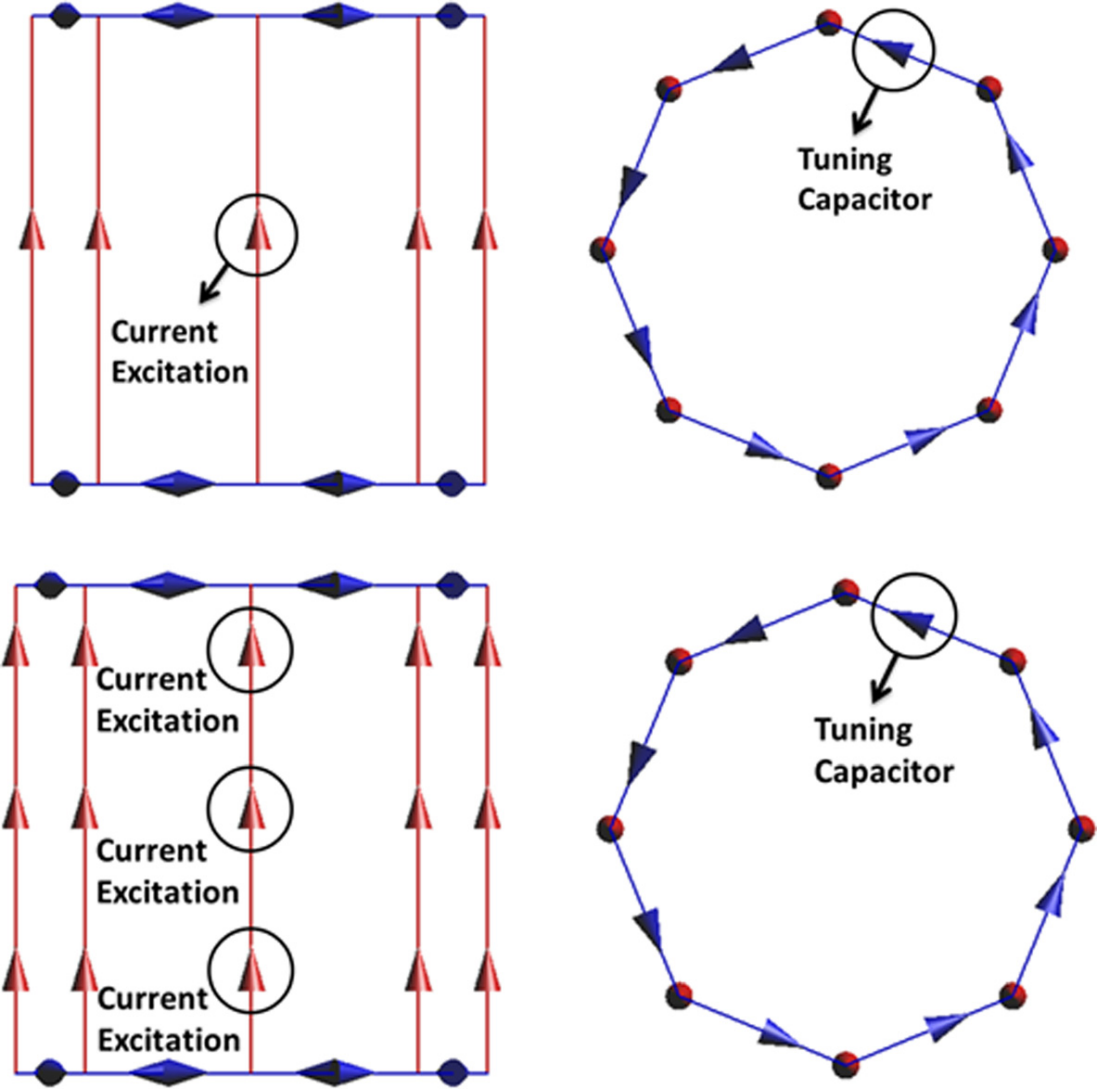
1.5-Tesla RF Coil (Top) and 3-Tesla RF Coil (Bottom) Models in SEMCAD.

**Figure 2:**
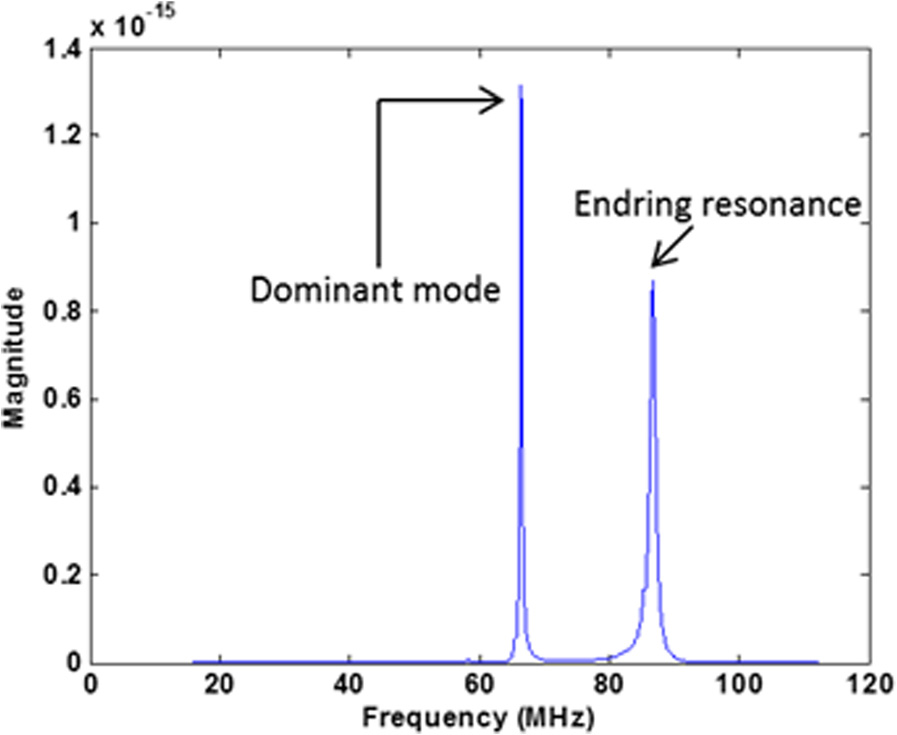
The power spectrum of 1.5-Tesla MRI RF coil excited by broadband signal with end ring tuning capacitance = 7.2 pF.

**Figure 3:**
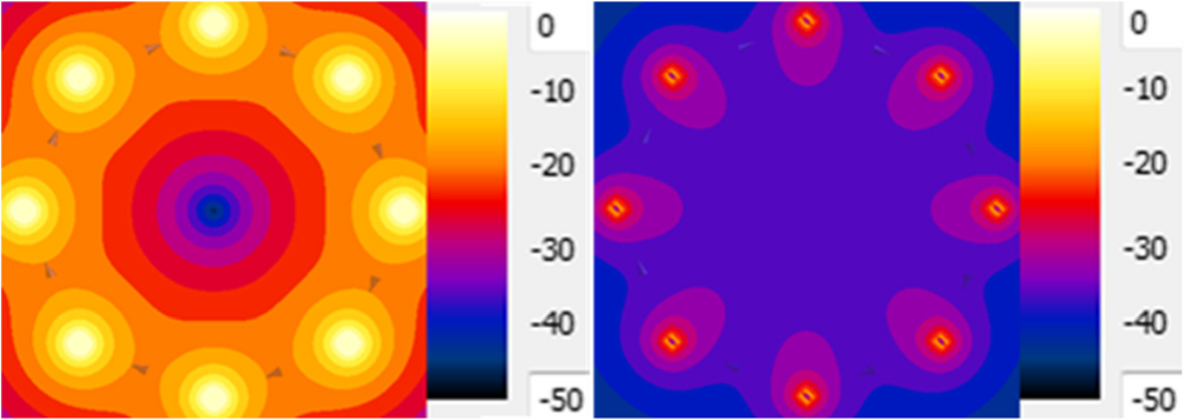
Electric Field (Left) and Magnetic Field (Right) distributions at center plane of MRI RF Coil.

To model the clinical relevant RF coils, two high pass RF transmit body coils were developed as shown in Figure 1. The top two models represent a 1.5-T RF coil and the bottom two models represent a 3-T RF coil. A physical coil is usually difficult to model because it requires the information of the detailed RF coil size, including the shape and size of the individual rungs and end-rings, and it takes much longer simulation time to reach the steady state of the simulation. It has been shown that using a non-physical coil could significantly reduce the simulation time while providing the same result as that from a physical coil. Thus, rather than modeling the exact physical coil, the non-physical coil are modeled in this study [10,15]. The non-physical RF coils are modeled using SEMCAD X [16]. The diameter of the RF coil is 63 cm. The height of the RF coil is 65 cm. The eight parallel red lines or the rungs are one dimensional line current sources. The blue lines or the end rings on top and bottom of the RF coils are capacitors which are also modeled as one dimensional line segments. The capacitance value is determined from several broadband simulations so that the second highest resonant frequency was adjusted to 64 MHz for 1.5-Tesla and 128 MHz for 3-Tesla systems [2]. The detailed steps are: set an initial capacitance value for all capacitors on end rings and add a broadband pulse signal on one single rung. The other seven rungs are modeled as zero ohm resistors. After the simulation is finished, the power spectrum is extracted. If the second highest resonant frequency is not at appropriate resonant frequencies, the capacitance value needs to be adjusted. After 3-To 5 broadband simulations, the second highest resonant frequency needs to be located at 64 MHz (for 1.5-T) as shown in Figure 2. From this study, the capacitance value for the end ring tuning capacitor is 7.2 pF for 1.5-Tesla RF coil and 1.3 pF for 3-Tesla RF coil. Figure 3 shows the electric and magnetic field distribution at the coil center inside the RF coil. The electric field is centrosymmetric and decreasing along the radial direction. The magnetic field is uniformly distributed. From Figure 2 and Figure 3, it is concluded that the RF coils are operating at the right resonant mode and the field patterns are also correct. Thus, the RF coils can be used for the following simulation studies.

**Figure 4:**
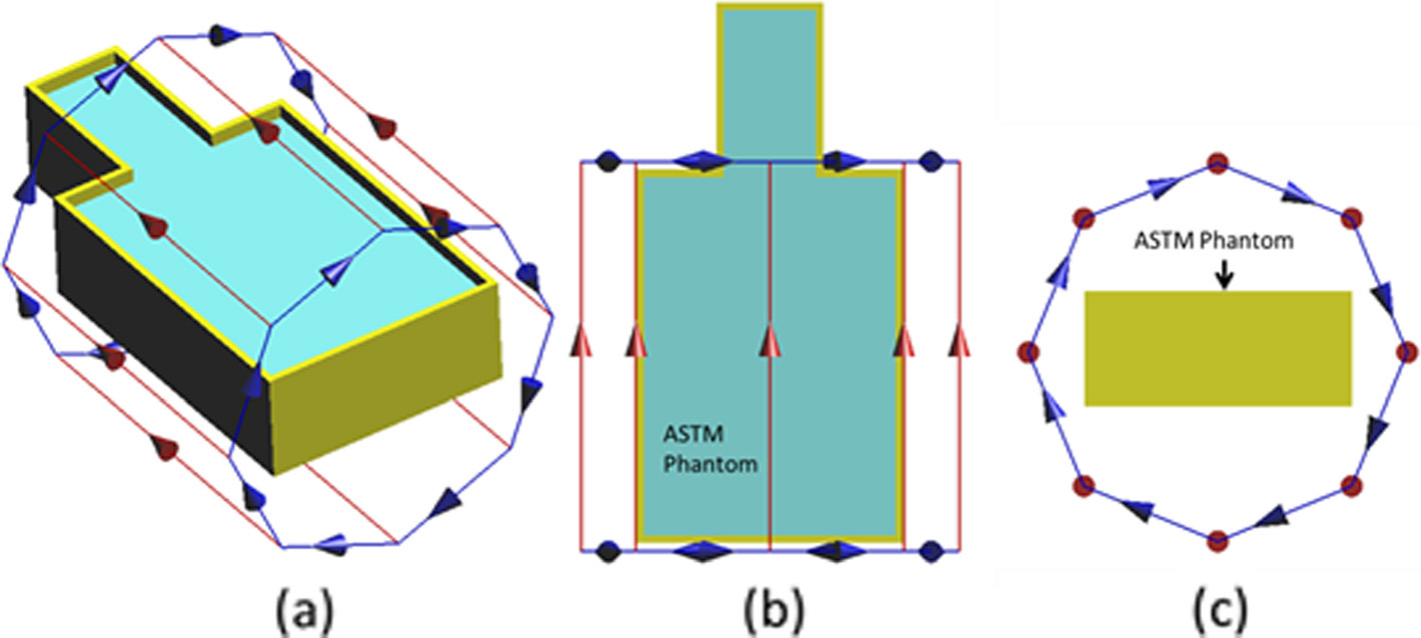
The illustration of the simulation setup to determine the maximum electric field location inside the ASTM phantom.

**Figure 5:**
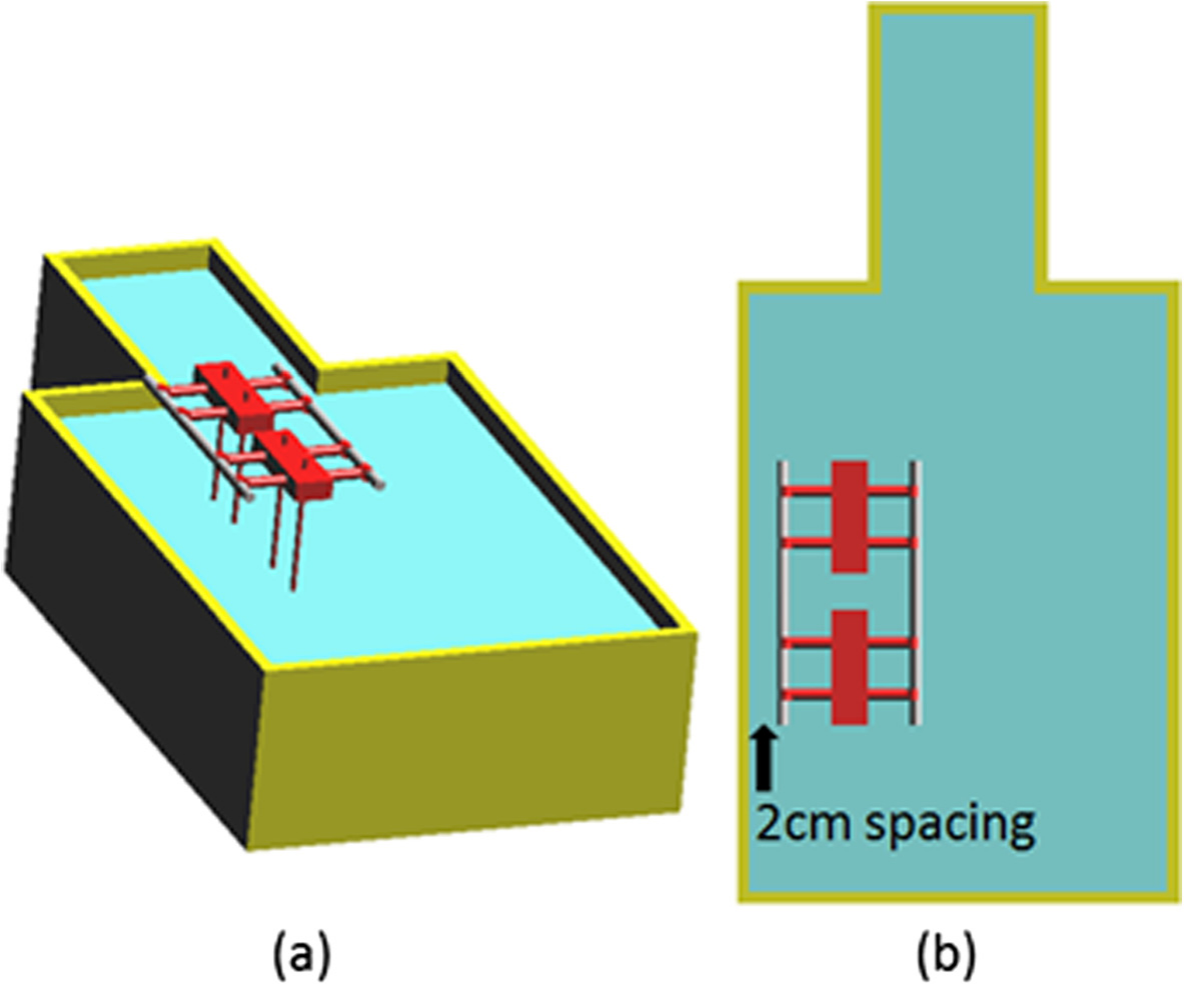
(a) Placement of external fixation device in the ASTM phantom; (b) 2 cm spacing between the outer bar and inner side wall in top view.

Once the RF coils were developed, the ASTM phantom was placed into each RF coil as shown in Figure 4. The center of the “trunk” section of the ASTM phantom was placed at the center of the RF coil. The bottom of the phantom was 23.85 cm above the lowest point of the RF coil. With this phantom placement, electromagnetic simulations were performed to determine the electric field distributions within the ASTM phantom. From simulations, it is determined that the maximum electric field locations are near the side walls of the phantom, along the vertical direction [10]. The higher electric field values in these regions are related to the region’s close spacing to the RF coil. Similar higher electric fields are also expected in these regions external to the ASTM phantom. Consequently, our external fixation devices are placed at these locations, as shown in Figure 5, so that the devices can be exposed to higher incident field both inside the ASTM phantom and outside the ASTM phantom.

**Figure 6:**
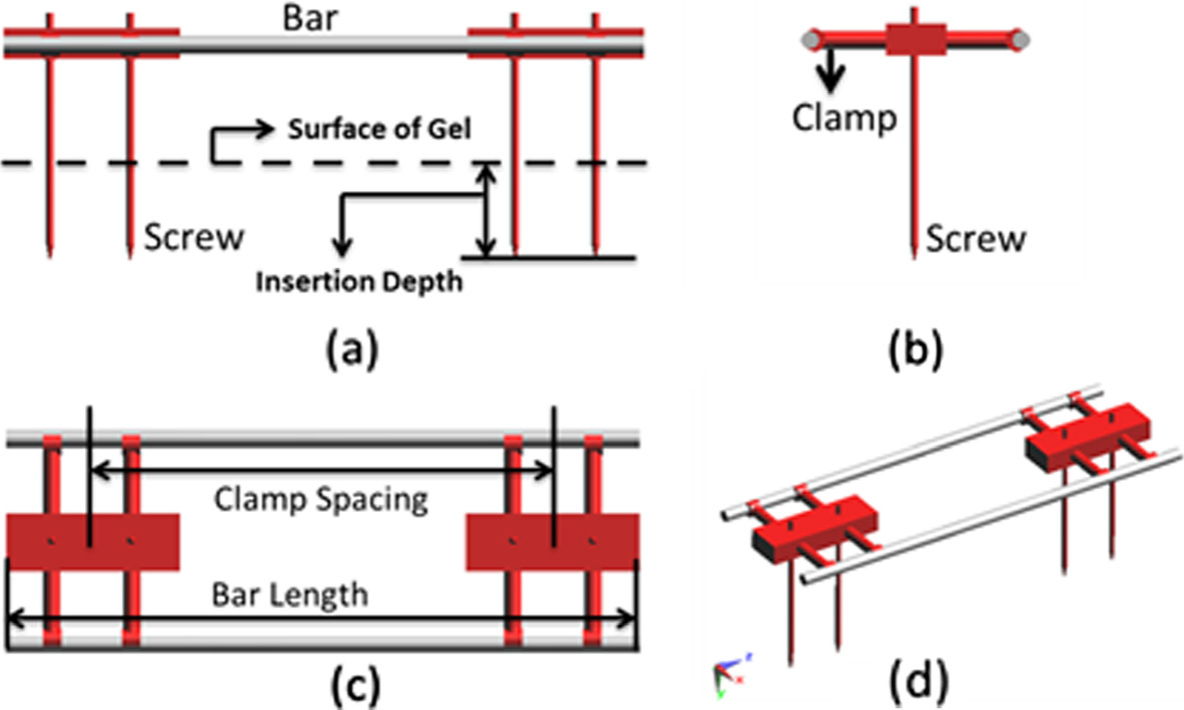
The structure of external fixation device with four pins, two main blocks and two connection bars.

The external fixation device model used in this study is comprised of three parts as shown in Figure 6. It consists of two metallic blocks to represent the clamps, two connectors or bars to represent the rods outside the body for rigid support, and four parallel long pins which are screwed into the bones during surgery. The metallic block has the dimension of 11.4 cm by 2 cm by 3.75 cm. The pin has a diameter of 0.5 cm and length of 16 cm. The connect bar has a diameter of 1.1 cm and can have four different length of 31.5 cm, 36.5 cm, 41.5 cm and 46.5 cm. When different bar lengths are used, the clamp spacing will be changed to 15 cm, 20 cm, 25 cm, and 30 cm. In all studies, the distance between the two connecting bar is set to be 5 cm.

The simulations were repeated with four different device pins insertion depths at 2 cm, 5 cm, 8 cm and 11 cm. Different set up for the simulations are summarized in Table 1. With deep pin insertion, the spacing between the connecting bar and the surface of the ASTM gel will be reduced as shown in Figure 5. There is a 2 cm spacing between the pins and inner side wall of the ASTM phantom shown in Figure 5(b). Three different bar materials are used in this study are 1) carbon-fiber with a relative dielectric constant of ε_r_ = 10 and electric conductivity of σ = 5.7e6 S/m, 2) plastic glass with a relative dielectric constant of ε_r_ = 4.4 and conductivity of σ = 0 S/m, and 3) perfect electric conductor (PEC) bar. The ASTM phantom consists of a plastic box whose relative dielectric constant is 3.7 and electric conductivity of 0 S/m. The gelled-saline has a relative dielectric constant of ε_r_ = 80.38 and conductivity of σ = 0.448 S/m [4]. The pins and main blocks of the device are modeled as perfect electric conductor.

**Table 1:** Electrical and thermal properties for different materials used in simulations

	**Permittivity**	**Electrical conductivity(S/m)**	**Thermal conductivity(W/m/K)**	**Heat capacity(J/kg/K)**	**Density(kg/m3)**
ASTM Phantom GEL	80.38	0.448	0.42	4160	1000
ASTM Phantom Shell	3.7	0	0.2	1000	1000
Device Bar (carbonfiber)	10	5600000	7	400	1000
Device Bar (PEC)	\	\	7	400	8000
Device Bar (plastic glass)	1	4.4	0.2	1000	1000
Device Other Parts (PEC)	\	\	7	400	8000

Electromagentic simulations are first conducted to determine electromagnetic energy deposition near the pins of the devices. Then, thermal simualtions are carried out to estimate the temperature rise near the tips. Based on the energy deposition near the device tips, the temperature rise in ASTM phantom is calculated using the heat transfer equation given as:

(1) $$\mathrm{\rho c}\frac{\partial T}{\partial t}=\nabla \cdot \left(k\nabla T\right)+\sigma {\left|E\right|}^{2}$$

where *ρ* is the gel density, c is the specific heat. The thermal conductivity k for ASTM plastic box, ASTM gelled-saline, and the device are 0.2 W/m/K, 0.42 W/m/K and 7 W/m/K, respectively. The specific heat capacity for gelled-saline is 4160 J/kg/K [4]. For plastic box and device, the spcific heat capacity is 1000 J/kg/K. The electric and thermal properties of the materials in this study is listed in Table 2.

**Table 2:** Device configurations and bar materials used in simulation study

**Device configuration ID**	**Clamp spacing(cm)**	**Insertion depth(cm)**	**Bar material**
1	15	2	Carbon Fiber/PEC/Plastic Glass
2	15	5	Carbon Fiber/PEC/Plastic Glass
3	15	8	Carbon Fiber/PEC/Plastic Glass
4	15	11	Carbon Fiber/PEC/Plastic Glass
5	20	2	Carbon Fiber/PEC/Plastic Glass
6	20	5	Carbon Fiber/PEC/Plastic Glass
7	20	8	Carbon Fiber/PEC/Plastic Glass
8	20	11	Carbon Fiber/PEC/Plastic Glass
9	25	2	Carbon Fiber/PEC/Plastic Glass
10	25	5	Carbon Fiber/PEC/Plastic Glass
11	25	8	Carbon Fiber/PEC/Plastic Glass
12	25	11	Carbon Fiber/PEC/Plastic Glass
13	30	2	Carbon Fiber/PEC/Plastic Glass
14	30	5	Carbon Fiber/PEC/Plastic Glass
15	30	8	Carbon Fiber/PEC/Plastic Glass
16	30	11	Carbon Fiber/PEC/Plastic Glass

## Results and discussion

The ASTM standard requires an RF field producing a sufficient whole body averaged SAR at 2 W/Kg for approximately 15 min for temperature rise measurement. Thus, in all following discussion for both 1.5-Tesla and 3-Tesla RF coils, the whole-body (WB) averaged SAR of 2 W/Kg is used to normalize the results. The WB averaged SAR is defined by the total absorbed power by the phantom gel divided by the total mass of the phantom gel.

**Figure 7:**
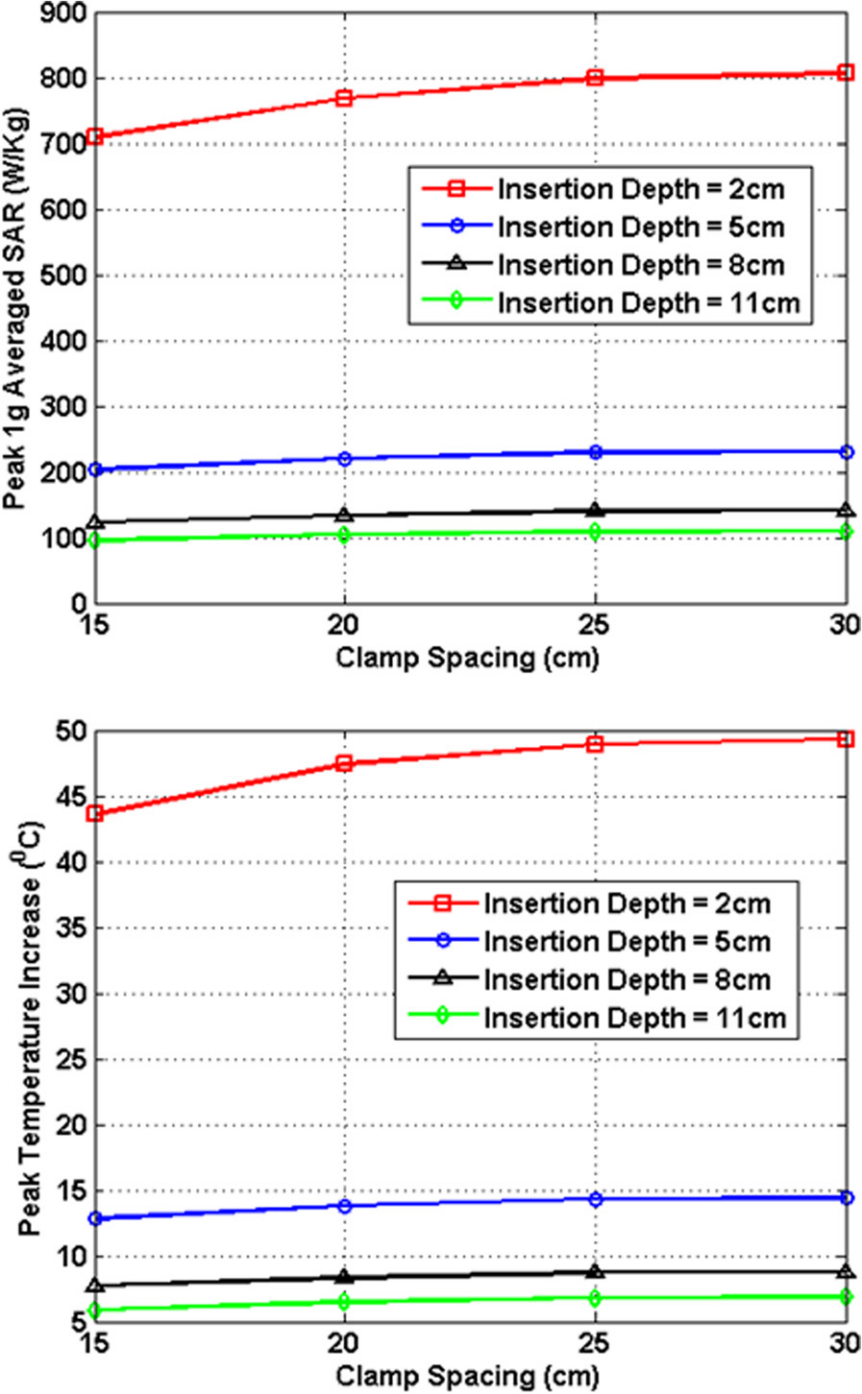
RF induced peak 1 g averaged SAR (Top) and maximum temperature rise after 15 min MRI scan (Bottom) for carbon fiber bar in 1.5-T MRI.

**Figure 8:**
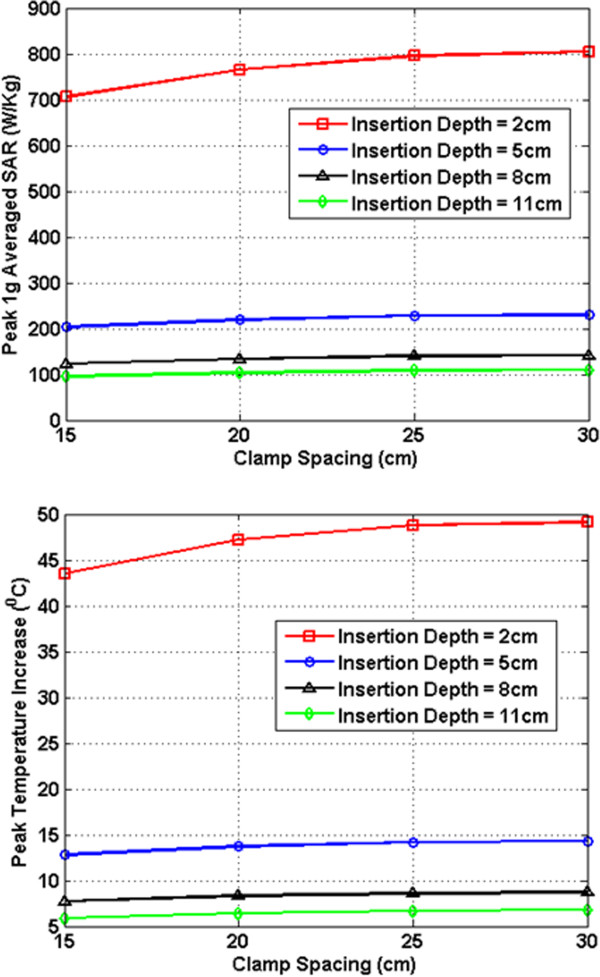
RF induced peak 1 g averaged SAR (Top) and maximum temperature rise after 15 min MRI scan (Bottom) for PEC bar in 1.5-T MRI.

**Figure 9:**
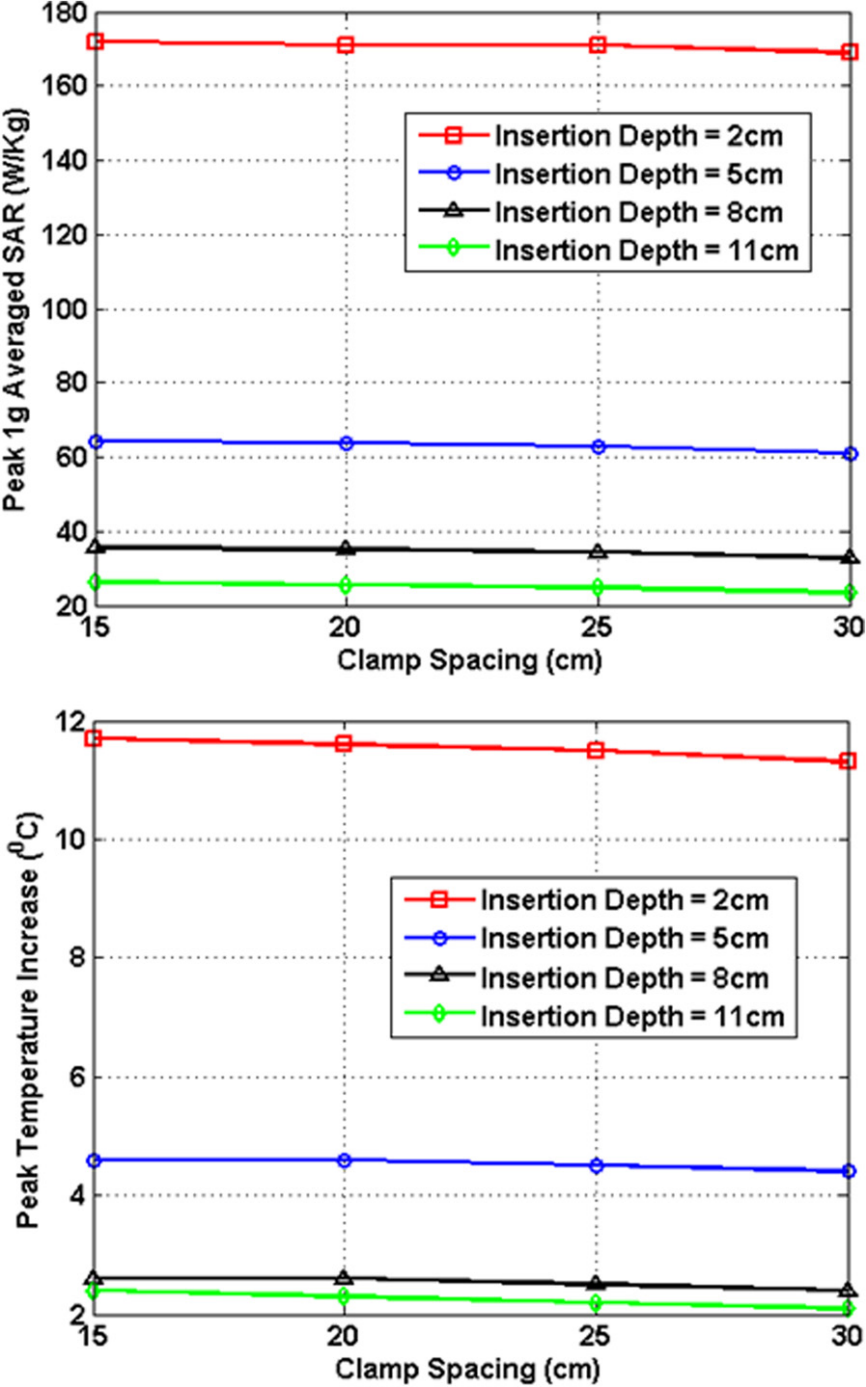
RF induced peak 1 g averaged SAR (Top) and maximum temperature rise after 15 min MRI scan (Bottom) for plastic glass bar in 1.5-T MRI.

**Figure 10:**
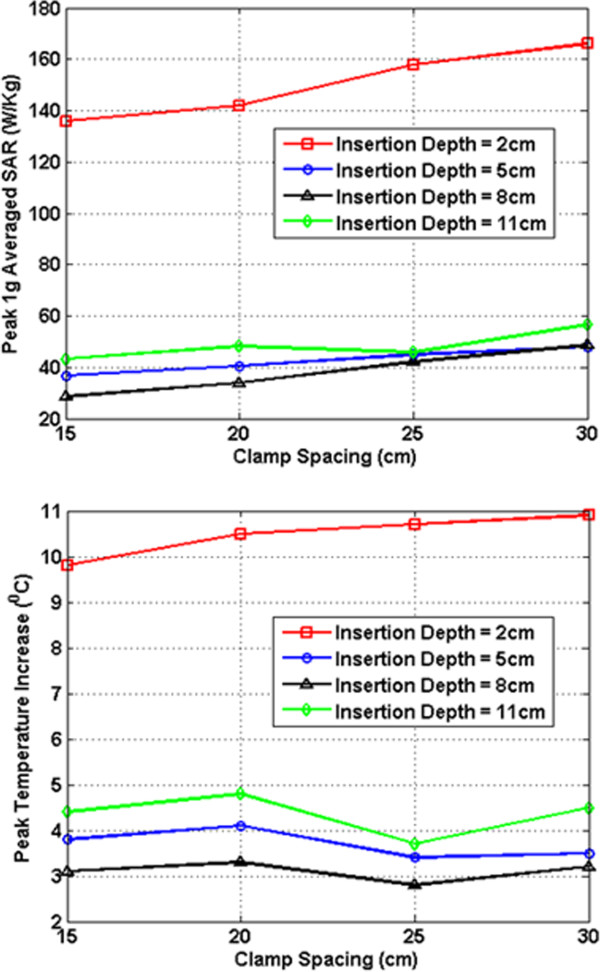
RF induced peak 1 g averaged SAR (Top) and maximum temperature rise after 15 min MRI scan (Bottom) for carbon fiber bar in 3-T MRI.

**Figure 11:**
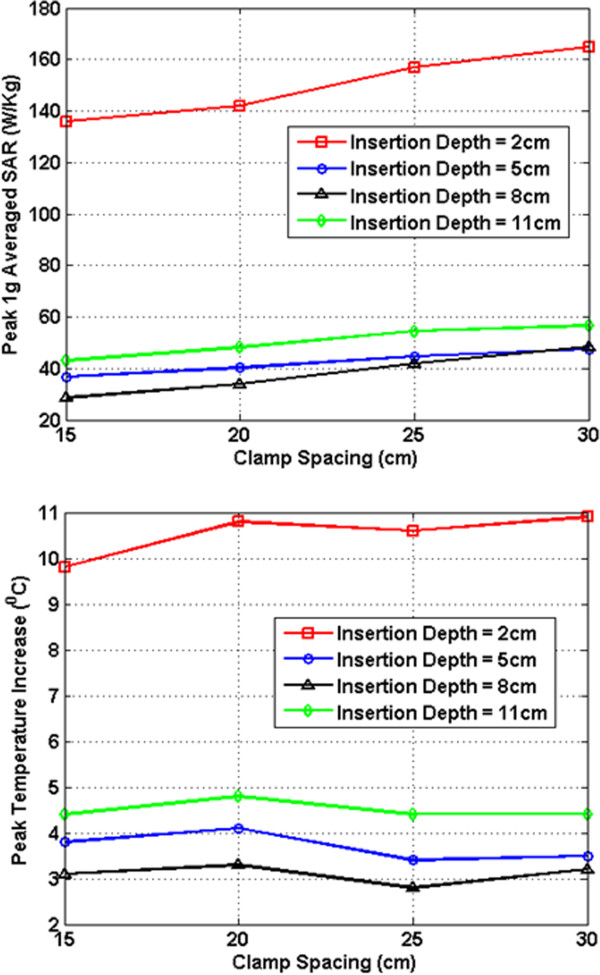
RF induced peak 1 g averaged SAR (Top) and maximum temperature rise after 15 min MRI scan (Bottom) for PEC bar in 3-T MRI.

**Figure 12:**
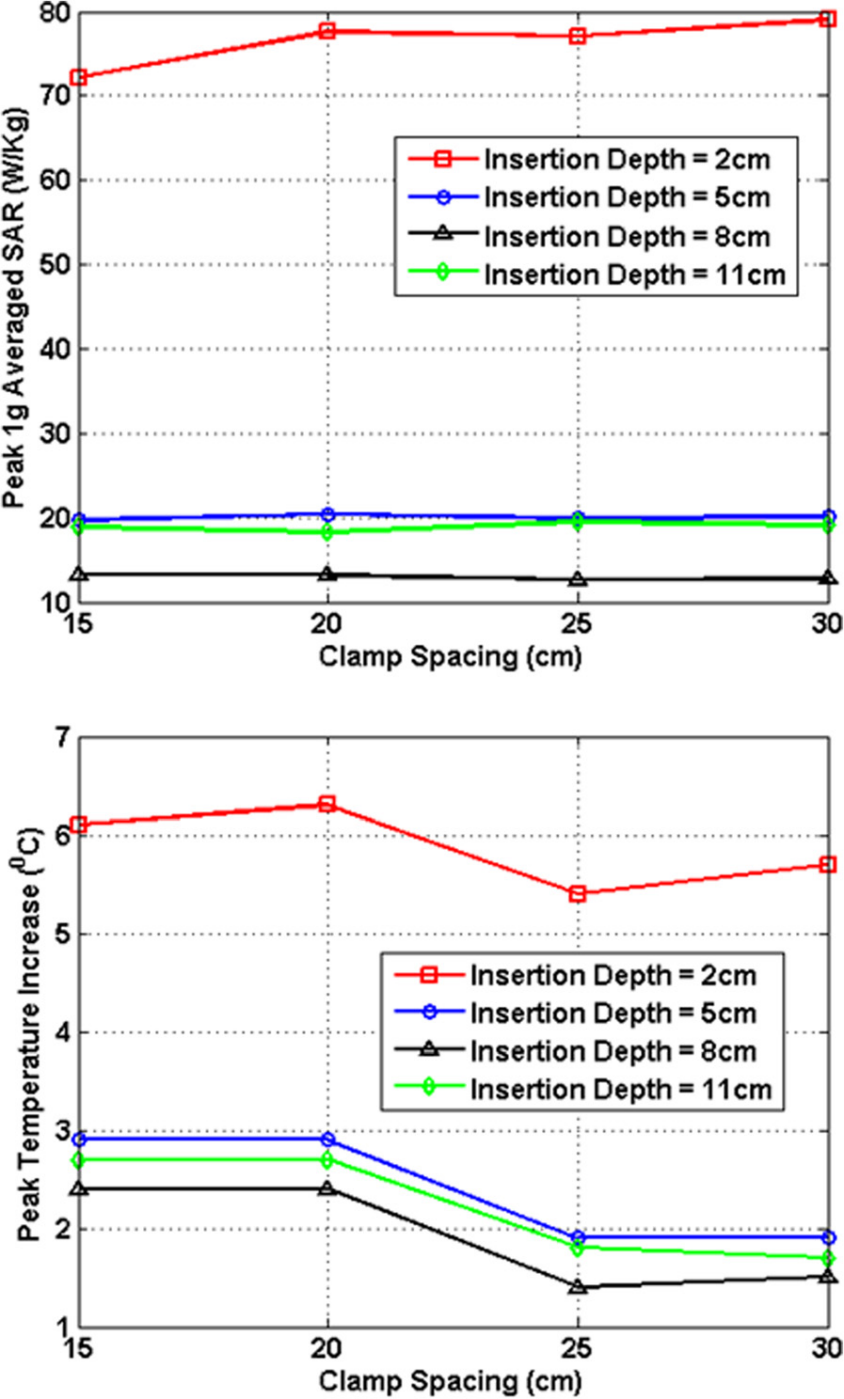
RF induced peak 1 g averaged SAR (Top) and maximum temperature rise after 15 min MRI scan (Bottom) for plastic glass bar in 3-T MRI.

In Figures 7, 8, 9, 10, 11, 12, the maximum 1 g averaged SAR in the ASTM phantom and the temperature rises near the device pins are shown for both 1.5-T and 3-T MRI systems. The temperature rise data are recorded for 15 minutes of MRI RF scan time. The results are listed in six groups with different bar materials and MRI systems. In each group, e.g. 1.5-T MRI environment and bar material as carbon-fiber, the 1 g averaged SAR and temperature rise data are shown with different clamp spacing (15 cm, 20 cm, 25 cm, and 30 cm). The four different curves in the plot represent four different insertion depth values: 2 cm, 5 cm, 8 cm, and 11 cm, respectively.

In the results shown in Figures 7, 8, 9, 10, 11, 12, all peak 1 g averaged SAR results correlate very well with the temperature rise results since the temperature rise is related to power deposition inside ASTM phantom. Higher peak 1 g averaged SAR value means higher local power loss around the pin. This will result in higher surface heating from the device pin and larger temperature rise is expected.

In 1.5-T MRI system, when the device bar is made by carbon fiber, as insertion depth increases, the temperature rise decreases dramatically. When clamp spacing increases, the temperature rise increases slightly. When the device bar is made of PEC, the temperature rise has very similar trend and similar value as those for carbon fiber bar. Therefore, in 1.5-Tesla MRI coil the carbon fiber device bar can be modeled as PEC to simplify modeling. In Figure 9, the bottom figure shows the temperature rise results using plastic glass bars. Structures with plastic glass bars have much less heating than those devices with carbon fiber bar and PEC bar in 1.5-Tesla MRI coil since the plastic glass bar is an electrical insulator. Such insulator will not induce additional energy deposition to the pins for the external fixation devices.

In 3-T MRI system, the temperature rise turns out to be much less than in 1.5-T MRI for the specific configurations studied here. For example, the worst-case temperature rise (30 cm pin spacing and 2 cm insertion depth) after 15 minutes the MRI scan is 11 degree in 3-T MRI compared with 59 degree in 1.5-T MRI. Similarly, for the 3-T MRI system, PEC bar is also a good approximation for carbon fiber bar. Both of them have very similar heating results. It is also found that, in 3-T MRI, when insertion depth increases, the temperature rise no longer keeps decreasing. The heating for 2 cm insert depth structure is higher than those from other three insertion depths. However, temperature rises for 5 cm, 8 cm and 11 cm inserting depth structures are comparable to each other. Similarly, in 3-T MRI, the device with plastic glass bar results in less surface heating than devices with a carbon fiber bar and PEC bar. However, the difference between temperature rises is not significant. This indicated that at 3-T, the major heating sources come from the induced energy on the clamps and the pins.

From these simulation results, it is determined that the heating of the external fixation device is related to the power loss at pin area which comes from the induced current flowing towards the tips of these pins. The total induced current comes from three parts: induced current on device bars, induced current on clamps, and induced current on pins. The primary component of electric field generated by MRI RF coil is parallel to the bar and perpendicular to the pins (this is especially true for 1.5-T system). Therefore, the induced current on the pins maybe negligible compared with the induced current coming from the other parts of the device. It is also observed that when the pin spacing increases, the induced current on the device bar will increase as long as the bar is conductive. When the connecting bar is insulated, there will be no induced current on the bar which results in less power loss in the pin, and thus lower temperature rise.

**Figure 13:**
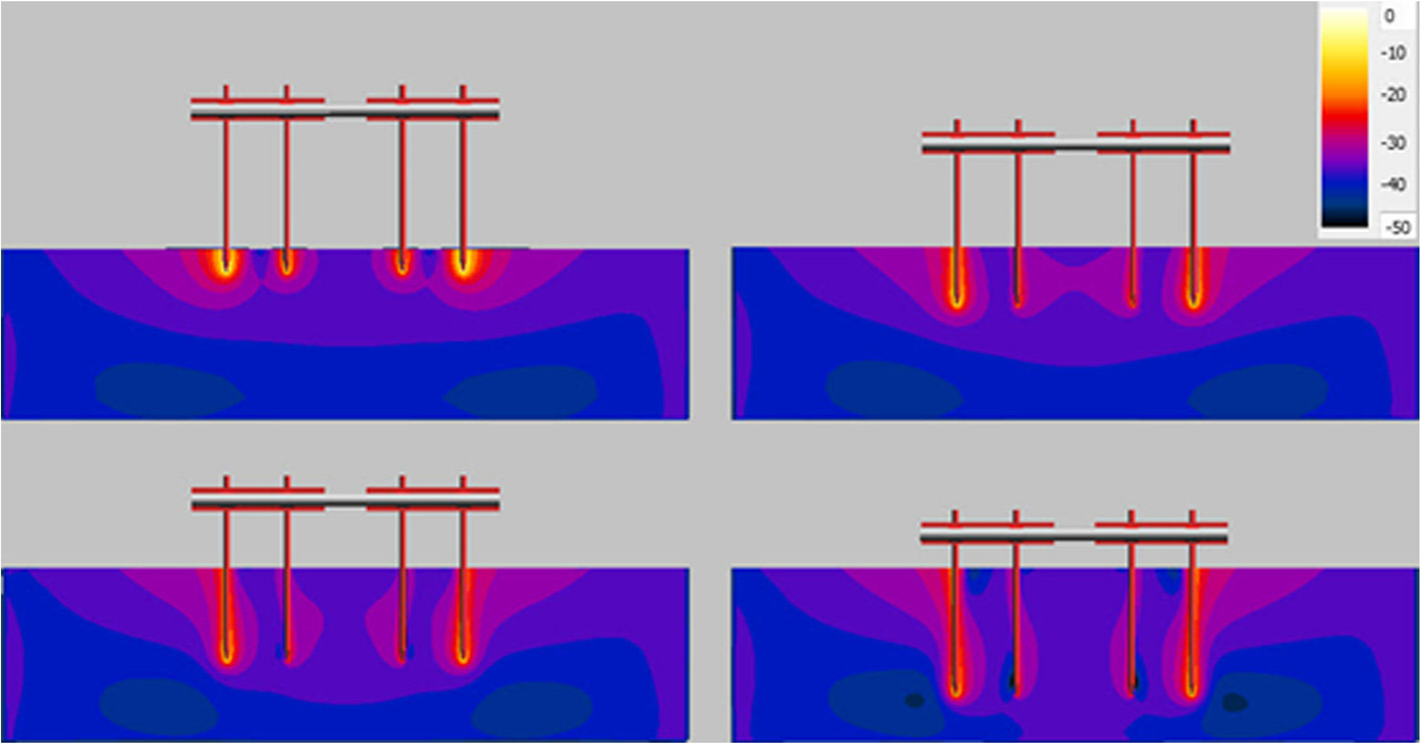
Power loss along the device pin with different insertion depth in 1.5-T MRI.

**Figure 14:**
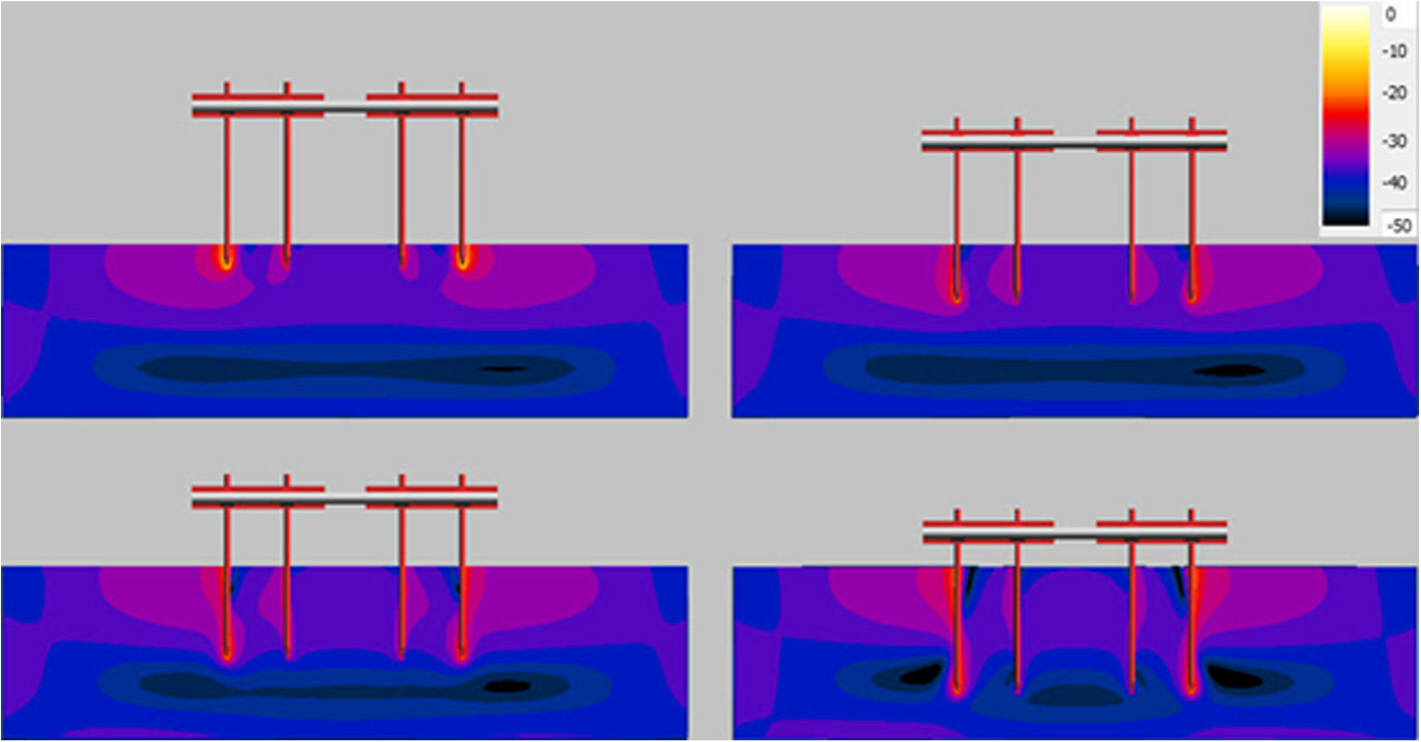
Power loss along the device pin with different insertion depth in 3-T MR.

The energy depositions inside the ASTM gel along different pins are shown in Figure 13 and Figure 14 for 15 cm clamp spacing construct at four different insertion depths. For the 1.5-T system, the highest energy deposition occurs at the tip of the outside two pins. The inner two pins have less energy deposition near them. When inserting depth increases, the heating effect tends to decrease dramatically. Because the ASTM gel is electrically lossy, as the induced energy travels towards the tip of the pin, longer insertion depth will lead to more power dissipated along the path. Thus, the shortest insertion depth can reserve most power and generate the worst-case heating.

For 3-T system, the frequency for the RF field is 128 MHz which is twice the frequency for the RF field in 1.5-T system. Consequently, the induced energy traveling along the pin inside the phantom gel will decay faster for 3-T system than that in 1.5-T system. As a result, the 2 cm insertion structure still has the highest heating. However, when the insertion depth goes beyond 5 cm, due to the quick power loss along the pin path, the devices induced heating become very small. Therefore, for deep pin insertion at 3-T, for the structures studied here, the pin tip heating is no longer of concern.

## Conclusions

Numerical simulation is used to study surface heating for external fixation devices in both 1.5-T and 3-T MRI coil. Typically, the shortest insertion depth and largest pin spacing with conductive bar will results in worst-case heating. The heating mechanism is explained using induced current along the device and power decay inside ASTM phantom.

## Competing interests

The authors declare that they have no competing interests.

## Authors’ contribution

YL carried most electromagnetic simulations, participated in thermal simulations, and drafted the manuscript. JS developed the CAD models and performed initial electromagnetic simulations. WK participated the data analysis. SQ performed part of the electromagnetic simulations and thermal simulations. WW participated the data analysis on both thermal simulation results. JC conceived of the study, participated all phases of the project, and helped to draft the manuscript. All authors read and approved the final manuscript.

## Disclaimer

The mention of commercial products, their sources or their use in connection with material reported herein is not to be construed as either an actual or implied endorsement of such products by the Department of Health and Human Services.
